# Supplementary material for: Altered Short Non-Coding RNA Landscape in the Hippocampus of a Mouse Model of CDKL5 Deficiency Disorder
Source: Biomolecules. 2025 Nov 17;15(11):1612. doi: 10.3390/biom15111612 (PMC12650334; doi:10.3390/biom15111612)
Supplement: Supplementary file 1 [file biomolecules-15-01612-s001.zip › biomolecules-3919694-supplementary Figure S1.pdf]

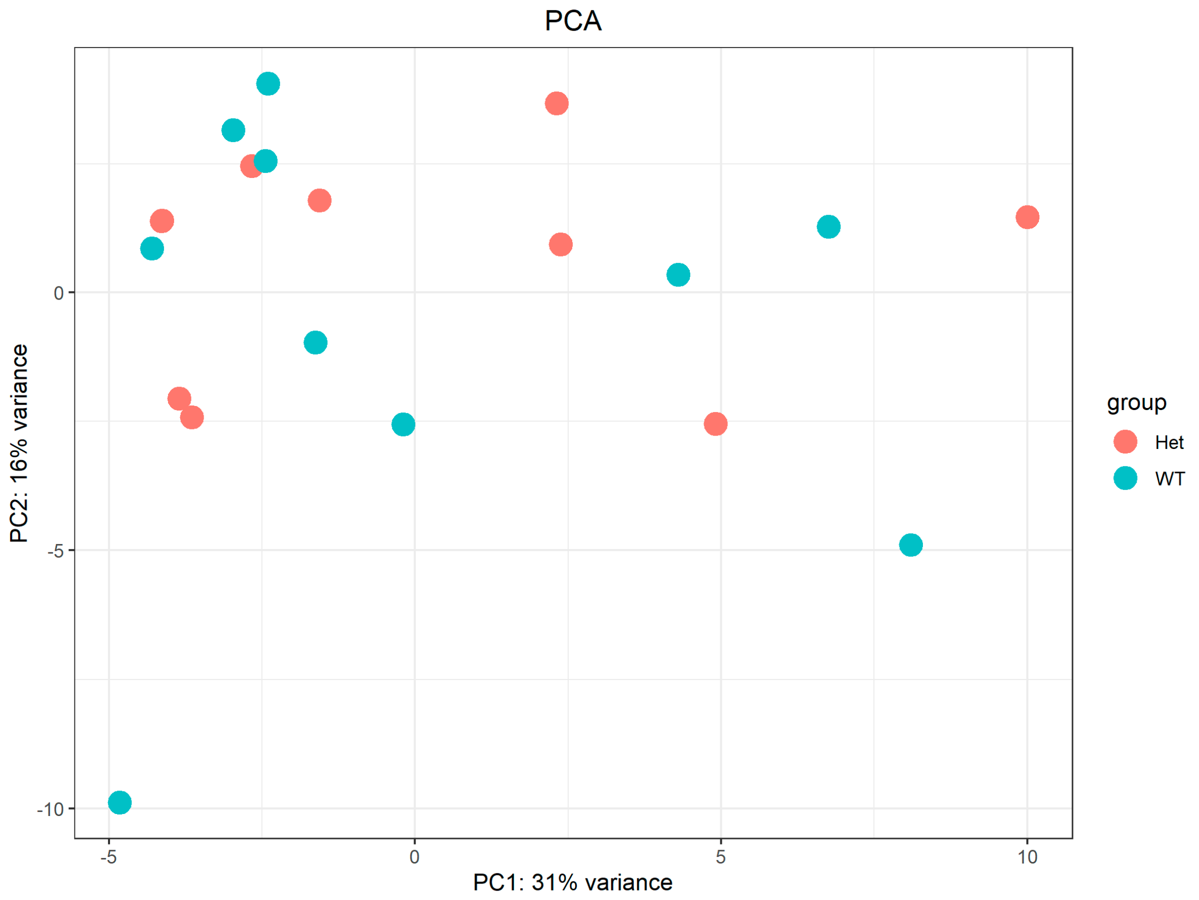

CDKL5 miRNA Group.PCA

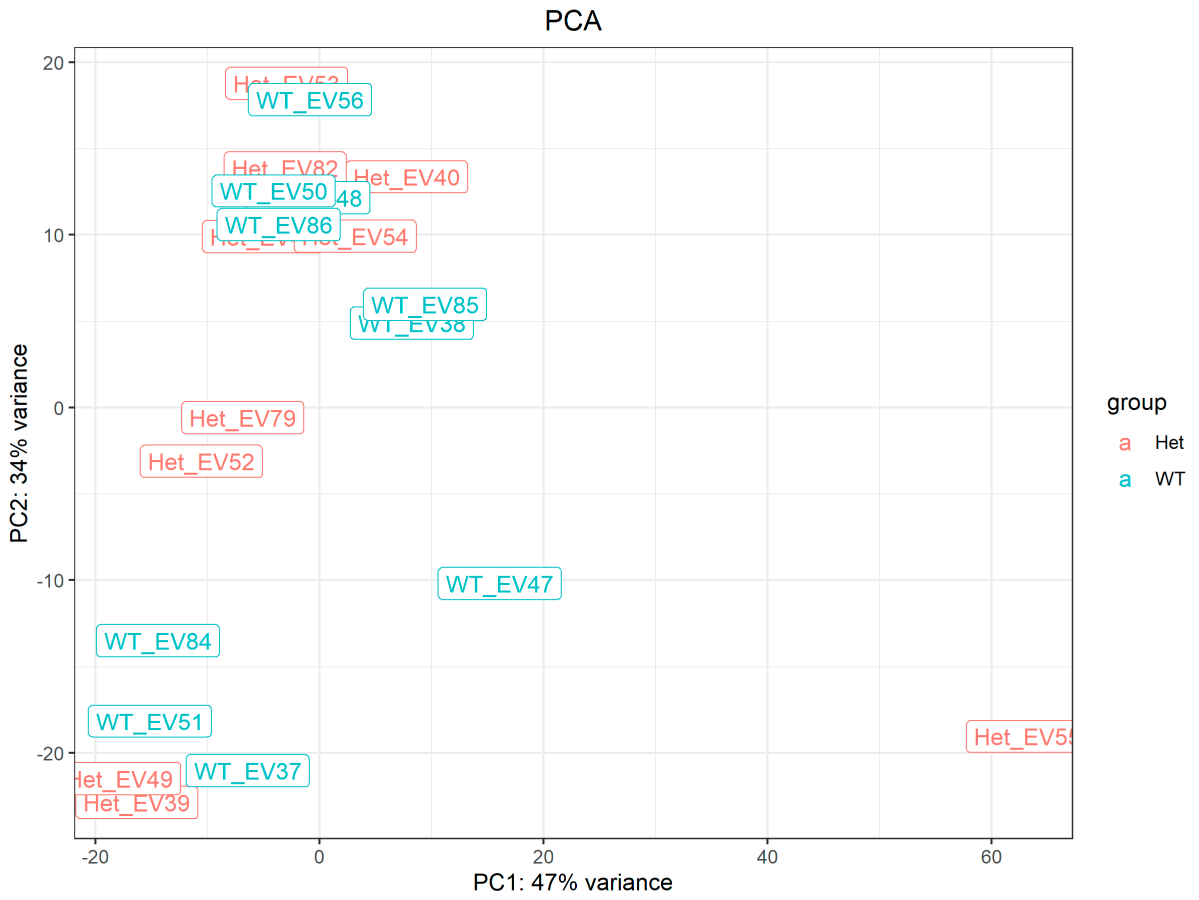

CDKL5 tRNA Group names.PCA

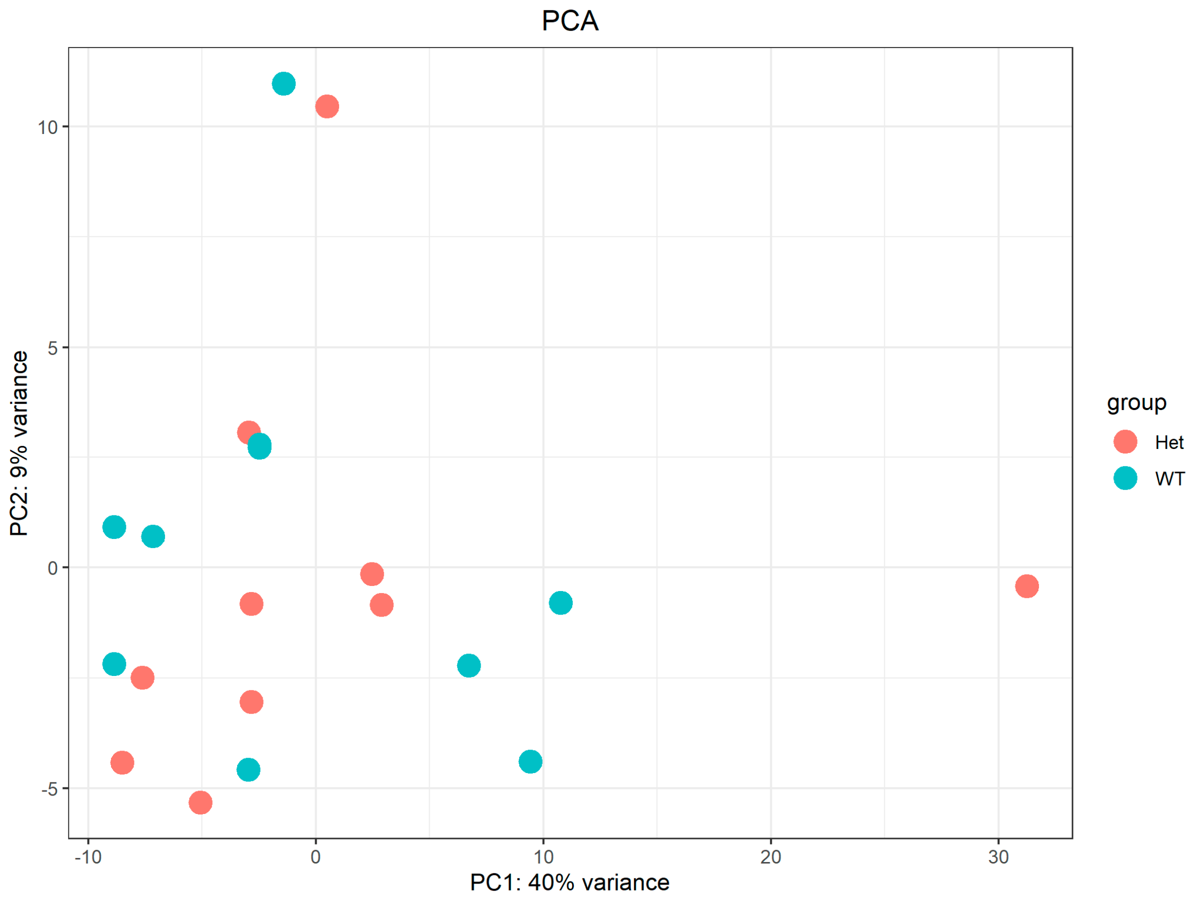

CDKL5 piRNA Group.PCA

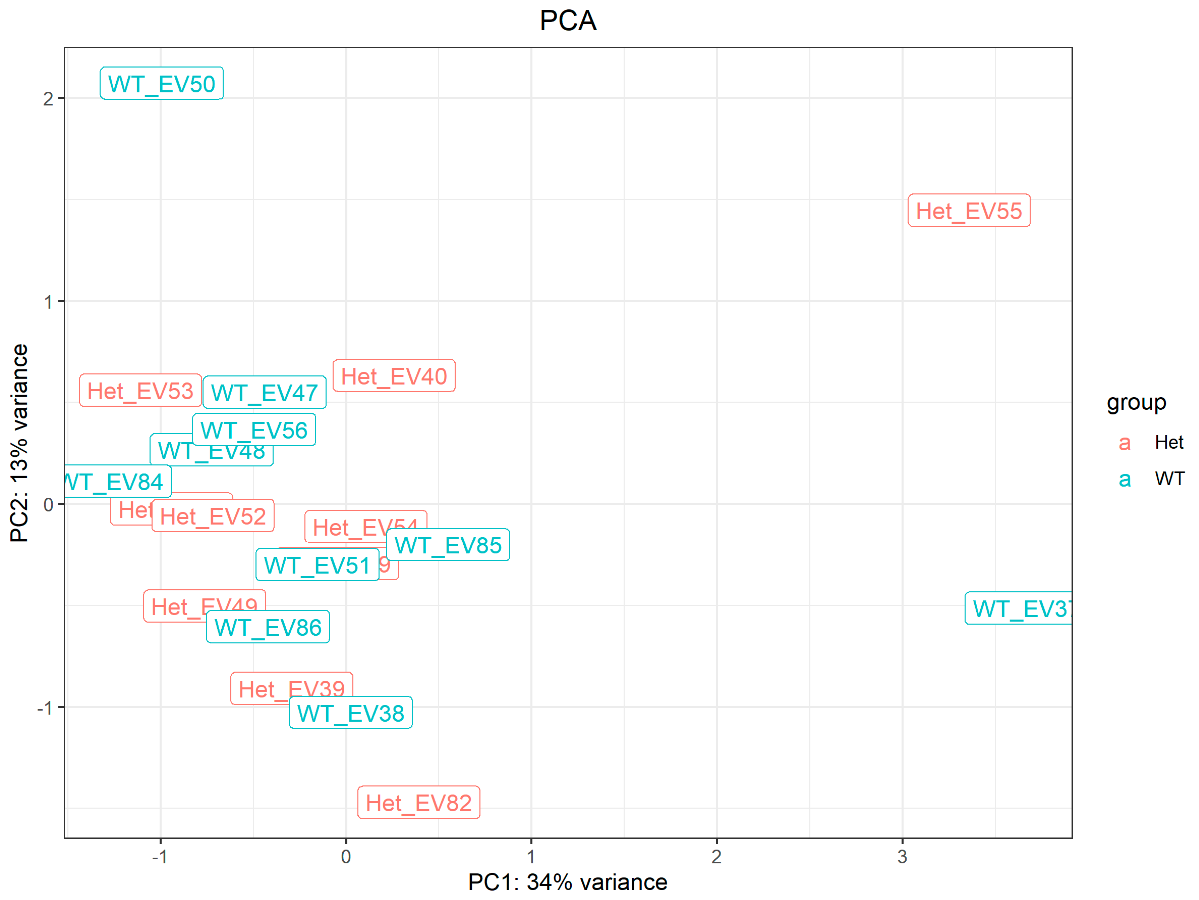

CDKL5 snoRNA Group names.PCA

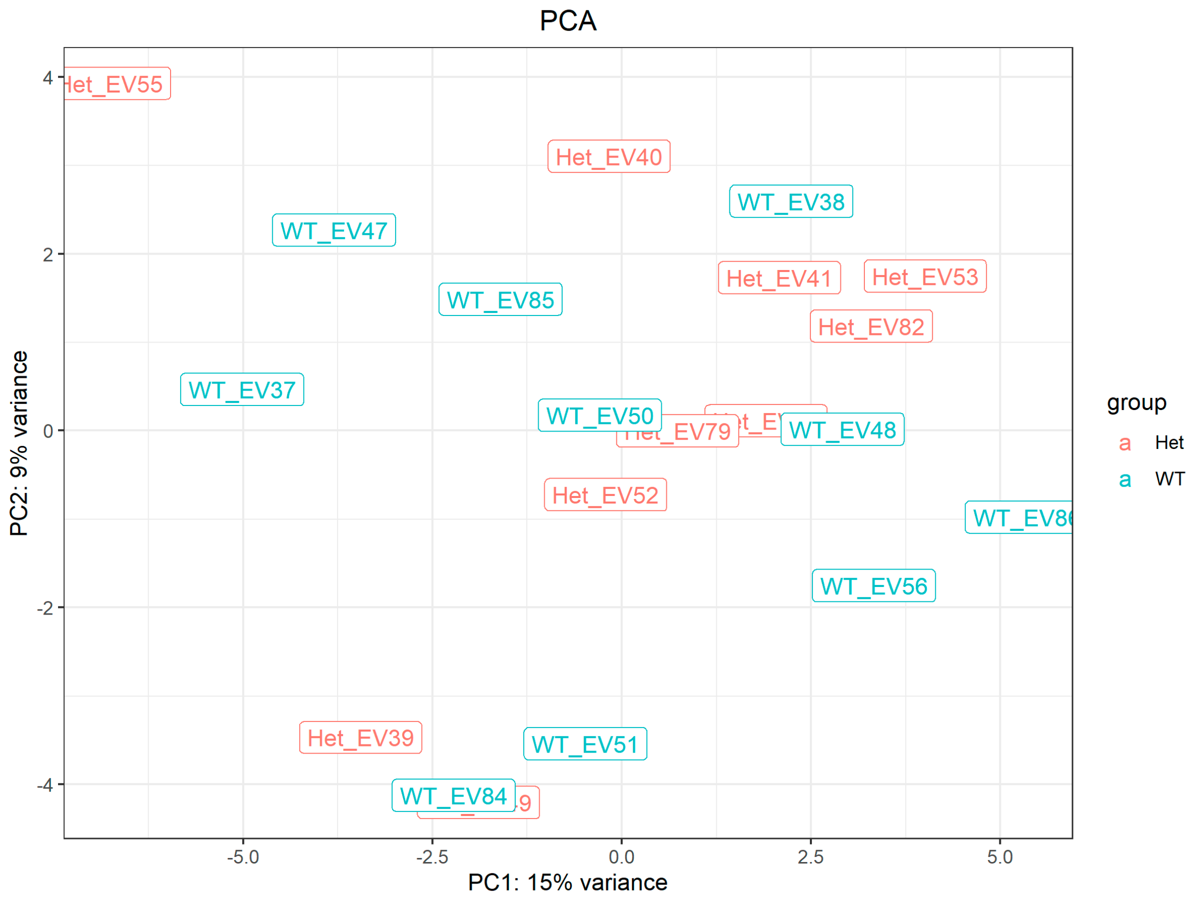

CDKL5 snRNA Group names.PCA
