# Supplementary material for: Altered Short Non-Coding RNA Landscape in the Hippocampus of a Mouse Model of CDKL5 Deficiency Disorder
Source: Biomolecules. 2025 Nov 17;15(11):1612. doi: 10.3390/biom15111612 (PMC12650334; doi:10.3390/biom15111612)
Supplement: Supplementary file 1 [file biomolecules-15-01612-s001.zip › biomolecules-3919694-supplementary Table S1.pdf]

miRNA	baseMean	log2FoldChange	lfcSE	stat	an adjusted pvalue
mmu-miR-150-3p	28,65974659	-0,451368029	0,158543392	-2,846968414	an adjusted p value
mmu-miR-1249-5p	2,726875813	-1,547457653	0,557286385	-2,776772761	an adjusted p value
mmu-miR-376c-5p	8,858822677	0,725451254	0,272062613	2,666486391	an adjusted p value
mmu-miR-200c-3p	59,91742085	-1,188744743	0,455638559	-2,608964318	an adjusted p value
mmu-miR-7046-3p	9,73600817	0,720668654	0,285430726	2,524846091	an adjusted p value
mmu-miR-1969	89,77757748	-0,307877115	0,125698728	-2,449325616	an adjusted p value
mmu-miR-377-3p	57,32917722	0,353477516	0,145182459	2,434712279	an adjusted p value
mmu-miR-6537-3p	4,836706957	-1,000529163	0,412480363	-2,425640719	an adjusted p value
mmu-miR-344-5p	4,49606164	0,931280213	0,386451696	2,409823071	an adjusted p value
mmu-miR-7000-5p	2,028133078	-1,513764438	0,63948141	-2,36717505	an adjusted p value
mmu-miR-505-3p	18,6157509	0,511247616	0,217762831	2,347726723	an adjusted p value
mmu-miR-467a-5p	756,8185166	-0,247638033	0,106398077	-2,327467199	an adjusted p value
mmu-miR-375-3p	1454,951035	-0,629178576	0,275947434	-2,280066772	an adjusted p value
mmu-miR-222-5p	136,7288226	0,214307501	0,094558213	2,266408125	an adjusted p value
mmu-miR-1943-5p	29,12718241	-0,546994734	0,242234228	-2,258123215	an adjusted p value
mmu-miR-145b	3,785314111	0,910606285	0,408356267	2,229930969	an adjusted p value
mmu-miR-6986-5p	8,151744414	0,627691283	0,288408623	2,176395684	an adjusted p value
mmu-miR-101c	93,41044117	-0,347455311	0,162322335	-2,140526819	an adjusted p value
mmu-miR-7685-5p	3,699415019	-0,93049836	0,435314719	-2,137530207	an adjusted p value
mmu-miR-297b-5p	14,50910081	-0,525723871	0,246862461	-2,129622578	an adjusted p value
mmu-miR-6911-5p	3,114492955	1,057790743	0,500498717	2,113473436	an adjusted p value
mmu-miR-5624-3p	1,532224774	-1,564775404	0,75288711	-2,078366575	an adjusted p value

mmu-miR-3473e	8,316233368	0,865771549	0,421731684	2,052896623	an adjusted p value
mmu-miR-215-3p	0,690127054	-2,35911581	1,154142461	-2,044042125	an adjusted p value
mmu-miR-144-3p	18,86819601	-0,855843957	0,421263365	-2,031612593	an adjusted p value
mmu-miR-101a-5p	9,276342218	-0,576659421	0,284516137	-2,02680743	an adjusted p value
mmu-miR-6929-3p	10,87690987	-0,501512847	0,247664659	-2,024967345	an adjusted p value
mmu-miR-671-5p	297,4972421	-0,203880474	0,103429888	-1,971194949	an adjusted p value
mmu-miR-384-3p	3680,520776	0,240575554	0,122584251	1,962532316	an adjusted p value
